# Image-based ex-vivo drug screening for patients with aggressive haematological malignancies: interim results from a single-arm, open-label, pilot study

**DOI:** 10.1016/S2352-3026(17)30208-9

**Published:** 2017-11-15

**Authors:** Berend Snijder, Gregory I Vladimer, Nikolaus Krall, Katsuhiro Miura, Ann-Sofie Schmolke, Christoph Kornauth, Oscar Lopez de la Fuente, Hye-Soo Choi, Emiel van der Kouwe, Sinan Gültekin, Lukas Kazianka, Johannes W Bigenzahn, Gregor Hoermann, Nicole Prutsch, Olaf Merkel, Anna Ringler, Monika Sabler, Georg Jeryczynski, Marius E Mayerhoefer, Ingrid Simonitsch-Klupp, Katharina Ocko, Franz Felberbauer, Leonhard Müllauer, Gerald W Prager, Belgin Korkmaz, Lukas Kenner, Wolfgang R Sperr, Robert Kralovics, Heinz Gisslinger, Peter Valent, Stefan Kubicek, Ulrich Jäger, Philipp B Staber, Giulio Superti-Furga

**Affiliations:** a CeMM Research Center for Molecular Medicine, Vienna, Austria; b Department of Biology, Institute of Molecular Systems Biology, ETH Zurich, Zurich, Switzerland; c Allcyte, Vienna, Austria; d Department of Internal Medicine I, Division of Hematology and Hemostaseology, Medical University of Vienna, Vienna, Austria; e Clinical Institute of Pathology, Medical University of Vienna, Vienna, Austria; f Department of Laboratory Medicine, Medical University of Vienna, Vienna, Austria; g Department of Biomedical Imaging and Image-guided Therapy, Medical University of Vienna, Vienna, Austria; h Division of General Surgery, Department of Surgery, Medical University of Vienna, Vienna, Austria; i Department of Internal Medicine I, Division of Oncology, Comprehensive Cancer Center, Medical University of Vienna, Vienna, Austria; j Ludwig Boltzmann Cluster Oncology, Medical University of Vienna, Vienna, Austria; k Center for Physiology and Pharmacology, Medical University of Vienna, Vienna, Austria; l Christian Doppler Laboratory for Chemical Epigenetics and Anti-Infectives, Vienna, Austria; m Pharmacy Department, Vienna General Hospital, Vienna, Austria; n Ludwig Boltzmann Institute for Cancer Research, Vienna, Austria; o Unit of Laboratory Animal Pathology, University of Veterinary Medicine, Vienna, Austria; p Ludwig Boltzmann Institute for Cancer Research, Vienna, Austria

## Abstract

**Background:**

Patients with refractory or relapsed haematological malignancies have few treatment options and short survival times. Identification of effective therapies with genomic-based precision medicine is hampered by intratumour heterogeneity and incomplete understanding of the contribution of various mutations within specific cancer phenotypes. Ex-vivo drug-response profiling in patient biopsies might aid effective treatment identification; however, proof of its clinical utility is limited.

**Methods:**

We investigated the feasibility and clinical impact of multiparametric, single-cell, drug-response profiling in patient biopsies by immunofluorescence, automated microscopy, and image analysis, an approach we call pharmacoscopy. First, the ability of pharmacoscopy to separate responders from non-responders was evaluated retrospectively for a cohort of 20 newly diagnosed and previously untreated patients with acute myeloid leukaemia. Next, 48 patients with aggressive haematological malignancies were prospectively evaluated for pharmacoscopy-guided treatment, of whom 17 could receive the treatment. The primary endpoint was progression-free survival in pharmacoscopy-treated patients, as compared with their own progression-free survival for the most recent regimen on which they had progressive disease. This trial is ongoing and registered with ClinicalTrials.gov, number NCT03096821.

**Findings:**

Pharmacoscopy retrospectively predicted the clinical response of 20 acute myeloid leukaemia patients to initial therapy with 88·1% accuracy. In this interim analysis, 15 (88%) of 17 patients receiving pharmacoscopy-guided treatment had an overall response compared with four (24%) of 17 patients with their most recent regimen (odds ratio 24·38 [95% CI 3·99–125·4], p=0·0013). 12 (71%) of 17 patients had a progression-free survival ratio of 1·3 or higher, and median progression-free survival increased by four times, from 5·7 (95% CI 4·1–12·1) weeks to 22·6 (7·4–34·0) weeks (hazard ratio 3·14 [95% CI 1·37–7·22], p=0·0075).

**Interpretation:**

Routine clinical integration of pharmacoscopy for treatment selection is technically feasible, and led to improved treatment of patients with aggressive refractory haematological malignancies in an initial patient cohort, warranting further investigation.

**Funding:**

Austrian Academy of Sciences; European Research Council; Austrian Science Fund; Austrian Federal Ministry of Science, Research and Economy; National Foundation for Research, Technology and Development; Anniversary Fund of the Austrian National Bank; MPN Research Foundation; European Molecular Biology Organization; and Swiss National Science Foundation.

## Introduction

Genetic studies have identified several genomic alterations associated with the development of haematological malignancies. However, barriers remain in fully translating this genomic information into direct clinical benefit for patients. Current efforts to introduce personalised medicine in patients with cancer, which focus on genetic and molecular patient stratification, have produced varying results.^1^, ^2^, ^3^, ^4^, ^5^ In a pioneering study,^4^ which used each patient as their own control, 27% of patients with recurrent metastatic cancer of any kind had a 30% longer progression-free survival with treatment selected on the basis of genetic profiling than they did with their previous treatment. However, the SHIVA study,^5^ one of the first randomised trials of genomic-based precision medicine, did not show a benefit in progression-free survival for patients assigned to genome-based targeted treatment compared with treatment according to physician's choice in heavily pretreated patients with cancer. Genome-based therapy decisions are limited by our incomplete understanding of the relationship between cancer phenotype and genotype, and the complex genetics underlying cancer are the result of dynamic microevolutionary processes.^6^ For instance, whereas several studies have linked cytogenetic and molecular abnormalities with distinct clinical outcomes in acute myeloid leukaemia,^7^ accurate prediction of treatment response of individual patients with acute myeloid leukaemia to induction therapy remains challenging.^8^, ^9^, ^10^, ^11^, ^12^ Furthermore, patients with aggressive haematological malignancies, who have failed at least two lines of therapy, are often without further standard treatment options and have a poor prognosis.^13^ These patients will usually receive either best available therapy, supportive care, or will be enrolled in clinical trials. Therefore, dynamic approaches that measure drug responses in cancer cells derived from patient biopsies might complement such static genetic measurements. For example, ex-vivo chemosensitivity tests have been done in samples from patients with chronic or acute leukaemia and multiple myeloma,^14^, ^15^, ^16^, ^17^, ^18^, ^19^, ^20^, ^21^ in breast cancer-derived stable cell lines,^22^ in patient-derived xenografts in mice,^23^, ^24^ and in gut stem-cell-derived organoids.^25^, ^26^ These pioneering functional assays have provided proof of concept by showing that ex-vivo responses might match clinical response; however, these studies have not been integrated into clinical routine because of practical limitations and scarce proof of clinical benefit.^27^, ^28^, ^29^

**Research in context**

**Evidence before this study**

We did a systematic search of PubMed using the search terms (“functional screening” [Title/Abstract] OR “chemosensitivity test” [Title/Abstract] OR “chemoresistance test” [Title/Abstract] OR “drug profiling” [Title/Abstract] OR “drug response” [Title/Abstract]) AND (“leukemia” [Title/Abstract] OR “leukaemia” [Title/Abstract] OR “lymphoma” [Title/Abstract] OR “myeloma” [Title/Abstract] OR “hematologic” [Title/Abstract]). We did not restrict the search by date, language, or article type. We did one search before initiating this study on Sept 1, 2015, and we repeated this search on Oct 13, 2017. Several studies have shown the potential for retrospective patient stratification based on a variety of ex-vivo drug-response profiling techniques; however, no reports were found of studies in which patient treatment for haematological malignancies were adapted to ex-vivo drug-response profiling across large panels of drugs. We also searched ClinicalTrials.gov for published clinical trials using the search terms described above. This search retrieved only one other clinical trial (currently recruiting patients and with feasibility as endpoint), in which patient treatment for haematological malignancies is being adapted to the drug-response profiles of primary biopsies across at least 100 different drugs tested.

**Added value of this study**

To our knowledge, our study is the first prospective study showing feasibility and efficacy of ex-vivo drug-response profiling to guide personalised treatment selection across large panels of possible treatments for patients suffering aggressive haematological malignancies. We do so with a new image-based, drug-response profiling technique that we call pharmacoscopy, which uses high-throughput, automated, confocal microscopy; immunofluorescence; and single-cell image analysis.

**Implications of all the available evidence**

Our interim study results indicate that adapting treatment regimens of patients with aggressive haematological malignancies to pharmacoscopy is feasible, safe, and effective. More patients whose treatment protocols were selected by the haematological tumour board based on pharmacoscopy results had an overall response and had longer progression-free survival with pharmacoscopy than their previous treatment. Further studies with randomised trial designs and larger patient cohorts than our study are justified to further elucidate the clinical impact of our novel, image-based, ex-vivo drug-response profiling platform.

Here, we investigate the clinical impact of a newly developed technology platform that combines multiparametric immunofluorescence with high-throughput automated microscopy and single-cell image analysis, called pharmacoscopy.^30^ Pharmacoscopy enables tumour-cell specific quantification of biological parameters of millions of adherent and non-adherent individual cells with high sample efficiency, minimal sample manipulation, extensive automation, and fast turn-around times. We thus aimed to evaluate the feasibility of integrating pharmacoscopy into the clinic, and to assess clinical response in patients who received a treatment according to pharmacoscopy results as an individual healing attempt.

## Methods

### Study design and participants

For this single-arm, open-label, pilot study, we collected samples and clinical data from patients with late-stage haematological malignancies. Patients were eligible for inclusion if no further standard treatments or clinical trials were available for the patient; the patient had undergone at least two lines of previous therapy; the patient gave written informed consent; cancer cell-containing samples could be biopsied after written informed consent; the clinical decision was made by a board consisting of haematologists, pathologists, pharmacists, and molecular biologists; and candidate treatments identified by pharmacoscopy were clinically available and considered safe given the patient's health condition. Pharmacoscopy-guided therapy was provided to individual late-stage patients as an individual healing attempt, in accordance with European Union and Austrian named-patient use legislation. Ethical approval was granted by the Ethics Commission of the Medical University of Vienna (Ethik Kommission 1830/2015, 2008/2015, 1895/2015).

### Procedures

Mononuclear cells from bone marrow aspirates, peripheral blood, pleural effusion, ascites, or excised lymph node samples were purified using Ficoll density gradient (bone marrow, peripheral blood, pleural effusion, ascites; Axis-Shield, Oslo, Norway) or homogenised and filtered through a 70-μm mesh filter (lymph tissue). The resulting single-cell suspensions of mononuclear cells were seeded in 384-well imaging plates containing small compound libraries which were incubated overnight (18 h at 37°C and 5% CO_2_). For most patients, libraries included 139 different drugs in two concentrations and five technical replicates total (appendix p 9). Bone marrow samples for the retrospective acute myeloid leukaemia study came from frozen biopsies, whereas biopsies used for the prospective study were all freshly acquired and not stored frozen. A comparison of pharmacoscopy results from fresh and frozen material from the same biopsy showed good consistency in results (appendix p 2). Immunofluorescence staining, imaging by automated microscopy (Opera Phenix; Perkin Elmer, Waltham, MA, USA), image analysis (CellProfiler; Broad Institute of Harvard and Massachusetts Institute of Technology, Boston, MA, USA), and data analysis (Matlab; versions R2015a, R2015b, R2016a, R2016b, R2017a, R2017b) were done as described previously.^30^ The antibodies used to identify the target blast population were selected based on clinical pathology antigen expression assessment reports, and included CD3 (HIT3a), CD19 (HIB19), CD20 (2H7), CD79a (HM47), CD34 (4H11), CD117 (104ED2), and CD138 (DL-101; eBiosciences [Thermo Fisher, Waltham, MA, USA]). Relative blast fractions (RBFs) were calculated as the fraction of marker-positive viable cells after drug treatment divided by the average fraction of marker-positive viable cells measured in dimethyl sulfoxide (DMSO)-containing control wells. For hierarchical clustering of ex-vivo drug responses, all RBF values per patient and marker combination were first averaged over technical replicates and concentrations per drug, and subsequently normalised into pharmacoscopy scores via (1 – RBF)/max (1 – RBF). Thus, a pharmacoscopy score of 1 represents the strongest on target ex-vivo response, a pharmacoscopy score of 0 indicates no ex-vivo effect, and negative pharmacoscopy scores indicate ex-vivo chemoresistance.

**Figure 1:**
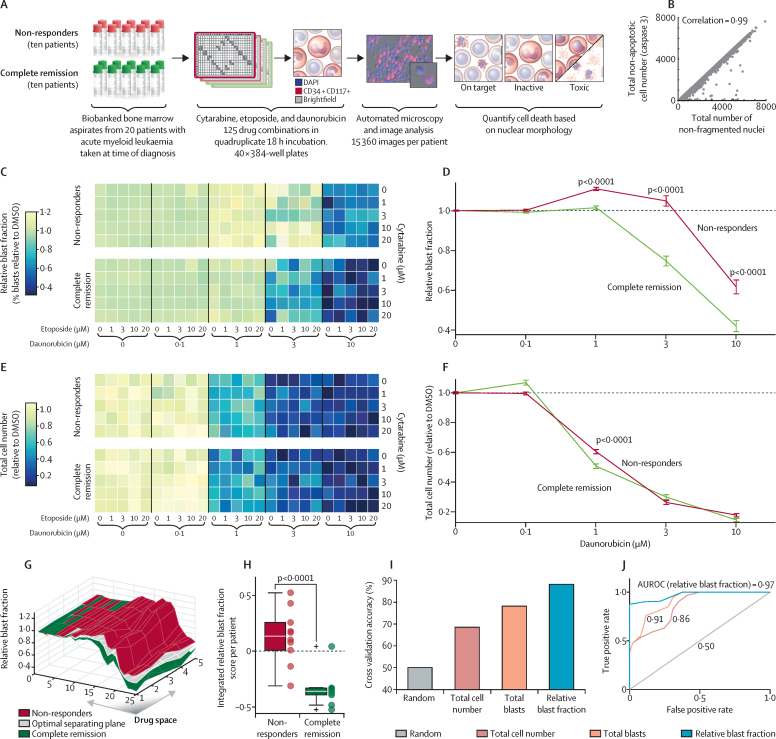
Pharmacoscopy and response to first-line acute myeloid leukaemia therapy (A) Schematic overview of the retrospective analysis using biobanked bone marrow samples of 20 patients with acute myeloid leukaemia taken before first-line treatment. (B) Comparison of two cell-death readouts: count of cells without activated caspase-3 and count of non-fragmented nuclei. Dots represent values from individual wells, for drug-screen results aggregated from three different patient samples. (C) Heatmap of the DMSO-relative fraction of CD34+ CD117+ blasts (C), and total cell number relative to DMSO (E), averaged for the non-responders and complete remission patient groups. Comparison of the DMSO-relative fraction of CD34+ KIT+ blasts (D) and total cell number (F) for the non-responders and complete remission groups, plotted as function of increasing concentrations of daunorubicin. Data are mean (SE) of patients. p values are for one-sided *t* test testing for reductions in complete remission group compared to non-responders. (G) Surface plot indicating the ideal separating hyperplane between the complete remission and non-responders groups. The drug space of 25 columns and five rows represents the same drug space as shown in (C) and (E). (H) Integrated RBF response scores per patient. Boxplots show distributions, dots are values for individual patients; crosses indicate datapoints that do not fall between the whiskers. (I) Cross-validation accuracies for integrated response scores for different cellular drug response readouts. (J) Average ROC curves over all cross-validation runs for different cell death readouts. AUROC values are indicated. (C–F) Assays done in technical quadruplicates for each of the 20 patient bone marrow samples. (I–J) Averaged classification results over 2025 cross-validation runs. AUROC=average area under ROC curve. DMSO=dimethyl sulfoxide. ROC=receiver operating characteristics.

To explore whether pharmacoscopy is predictive of clinical response, we designed a retrospective study using samples from patients with acute myeloid leukaemia collected before receiving standard first-line remission induction therapy (figure 1A). Roughly 60% of patients typically respond with complete remission to induction therapy,^31^ which consists of cytarabine combined with daunorubicin and etoposide.^32^, ^33^ Each patient sample was screened through a drug combination matrix of all three first-line drugs, consisting of 125 unique drug concentration combinations in four technical repeats. To determine the ex-vivo drug-induced cytotoxicity, we quantified the number of non-fragmented nuclei in each image after drug treatment. Drug-induced cell death based on nuclear morphology was measured after overnight drug incubation, and subpopulation specificity was assessed on the cells that stained positive for CD34 or KIT (CD117). Both markers are commonly present on leukaemic blasts in acute myeloid leukaemia.^34^ Because all patient samples contained a combination of blast cells and non-malignant cells, we calculated the RBF of drug-treated cells to compare on target drug-induced cytotoxicity with that of drug-induced cytotoxicity in blast-marker-negative cells (figure 1A). The RBF is thus defined as the fraction of viable blasts surviving drug treatment relative to the average fraction of viable blasts observed in negative control samples. Drug sensitivity per patient was integrated over the drug matrix by averaging the number of RBF datapoints above (scored with +1) or below (−1) the hyperplane that best separated responders from non-responders (figure 1G), weighted by the area under the receiver-operating characteristic curve (AUROC) of each corresponding concentration point in the drug matrix. We also compared clinical response with cytogenetic and molecular risk classification.

For the prospective study, eligible patients were assessed by the board (PBS, UJ, GIV, KM, CK, GH, IS-K, KO, WRS) and those who met inclusion criteria were tested by pharmacoscopy as outlined above. The markers used to identify the blast populations were selected individually for each patient based on their disease indication and clinical diagnostics. For the prospective study, on-target cytotoxicity was identified by calculating the RBF as in the acute myeloid leukaemia retrospective analysis, in which blast, in this context, now referred to any cancer cell. Thus, top-scoring drugs achieved the most specific reduction of the tumour-cell-enriched cell fraction ex vivo, while causing minimal cytotoxicity to the marker-negative healthy cells also present in the sample. The board then assessed the results, taking into account an individual patient's previous treatment outcomes to recommend the next treatment regimen. Patients assessed by the board, but who had further standard treatment options, were used as an observational cohort. Integration of data from both patient groups allowed us to test whether chemoresistance measured by pharmacoscopy (eg, ex-vivo survival of blast cells coinciding with death of non-malignant cells) is predictive of poor clinical response. To gain an overview of the complete dataset, we first set out to cluster the drug-response profiles. For this purpose, RBF values were normalised to pharmacoscopy scores; negative values indicate drug resistance (blast survival and non-malignant-cell death), and positive values indicate on-target chemosensitivity (blast death and non-malignant-cell survival; appendix p 5).

To account for the complicating fact that for most treatment regimens comprised of multiple drugs, ex-vivo testing was in fact done with single drug treatments, and that multiple and varying number of blast markers were measured in different patients depending on their clinical diagnostic results, we summed the relevant pharmacoscopy values over all drugs and markers per patient, resulting in an integrated pharmacoscopy (i-PCY) score. We quantified overall response as 1=progressive disease, 2=stable disease, 3=partial response, 4=complete remission, and determined the correlation with i-PCY.

### Outcomes

The primary outcome measure was the proportion of patients achieving progression-free survival, and the secondary outcome measure was the proportion of patients with an overall response (achieving either a complete remission or partial response). Progression-free survival was calculated as the time from the first day of treatment to the date of the first reported disease progression or relapse, initiation of a new (unplanned) anticancer treatment, or death as a result of any cause. Overall response was defined by achieving either complete remission or a partial response, defined by standard response definition guidelines.^35^, ^36^ For patients with lymphoma, responses were classified as complete remission, partial response, stable disease, or progressive disease according to the criteria proposed by the international working group on malignant lymphoma.^35^ For patients with leukaemia, responses were assessed following the response criteria defined by the recommendations of the European LeukemiaNet.^36^ All patients that were included in the prospective trial had uniform follow-up intervals of 4 weeks.

### Statistical analysis

The treatment was deemed to be of clinical benefit for the individual patient who has a progression-free survival ratio (progression-free survival on pharmacoscopy-guided therapy/progression-free survival on prior therapy) of 1·3 or higher. In such cases, we rejected the null hypothesis, defined as 15% or fewer patients having a progression-free survival ratio of 1·3 or higher. Thus, the individual patient was their own control. Comparisons of the overall response to previous treatment and pharmacoscopy-guided treatments were calculated using a one-sided McNemar's test for paired binomial data with continuity correction. The odds ratio (OR) could not directly be calculated as one of the discordant values (those patients who did respond to the most recent treatment, but who did not respond to pharmacoscopy-guided treatment) was equal to zero. We therefore calculated the overall response-associated OR using the standard calculation for contingency tables. Significance testing for progression-free survival differences was done using the log-rank (Mantel-Cox) test. All correlations are Pearson correlation coefficients. All other p values are two-tailed *t* tests, unless stated otherwise. Statistical analyses were done in GraphPad Prism (version 7), Matlab (versions R2015a, R2015b, R2016a, R2016b, R2017a, R2017b), and Microsoft Excel (version 2016).

The trial was registered at the ClinicalTrials.gov trial registry, number NCT03096821.

### Role of the funding source

The funders of the study had no role in study design, data collection, data analysis, data interpretation, or writing of the report. PBS and UJ had access to patient annotated clinical data, BS, GIV, and GS-F had access to the anonymised patient clinical data and correlated drug responses. The corresponding author had full access to all the anonymised results and final responsibility for the decision to submit for publication.

## Results

The retrospective acute myeloid leukaemia study to determine whether pharmacocopy is predictive of clinical response used 20 biobanked bone marrow samples; ten samples from patients achieving stable complete remission to induction therapy, and ten from non-responders to induction therapy.^32^, ^33^ The correlation coefficient (*r*) when examining the number of non-fragmented nuclei in each image after drug treatment was 0·99 with immunofluorescence against activated caspase-3 as a measure of cell death over samples from three patients, confirming the nuclear morphology readout (figure 1B). Bone marrow immunohistochemistry from clinical diagnostics confirmed the presence of CD34 and CD117 on blasts of all 20 patients. The 20 patients represented both sexes and diverse ages, had diverse genetic lesions and karyotypes, and blast fractions at time of sampling ranging from 30% to over 90% (appendix p 6). The clinical response to treatment in our retrospective cohort of patients with acute myeloid leukaemia only partially followed the cytogenetic and molecular risk classification (appendix pp 3, 6), with, for instance, all four patient who had a *FLT3-ITD* mutation in the non-responders group and both inv(16) patients in the complete remission group.

The RBF was significantly different between complete remission and non-responders groups, with significantly stronger on-target effects observed with ex-vivo daunorubicin treatment for the complete remission patient cohort (p<0·0001; figures 1C, 1D, appendix pp 3, 7). Conversely, population-averaged cytotoxicity measurements (total cell death) did not correctly stratify patients based on their clinical response (figures 1E, 1F), indicating the need for the relative drug sensitivity measurements. As expected, the integrated response score for drug sensitivity revealed good separation of responders and non-responders (figure 1H, appendix p 3). One particularly strong outlier was observed, complete remission in patient 10 for whom no ex-vivo response was measured. This discrepancy could not be attributed to differences in clinical parameters nor technical issues. Cross-validation by leaving out and reclassifying every possible combination of two patient samples, and calculation of the ideal hyperplane based on the remaining 18 samples, revealed an average classification accuracy of 88·1% for the RBF (figure 1I), and an average AUROC of 0·97 (figure 1J). Consistently, we observed reduced classification power for this cohort with population-averaged readouts: overall cell death, quantified by the total cell number, led to a classification accuracy of 68·5% (AUROC 0·86), and cell death of marker-positive cells, quantified as the total blasts, led to a classification accuracy of 78·1% (AUROC 0·91; figures 1I, 1J).

**Figure 2:**
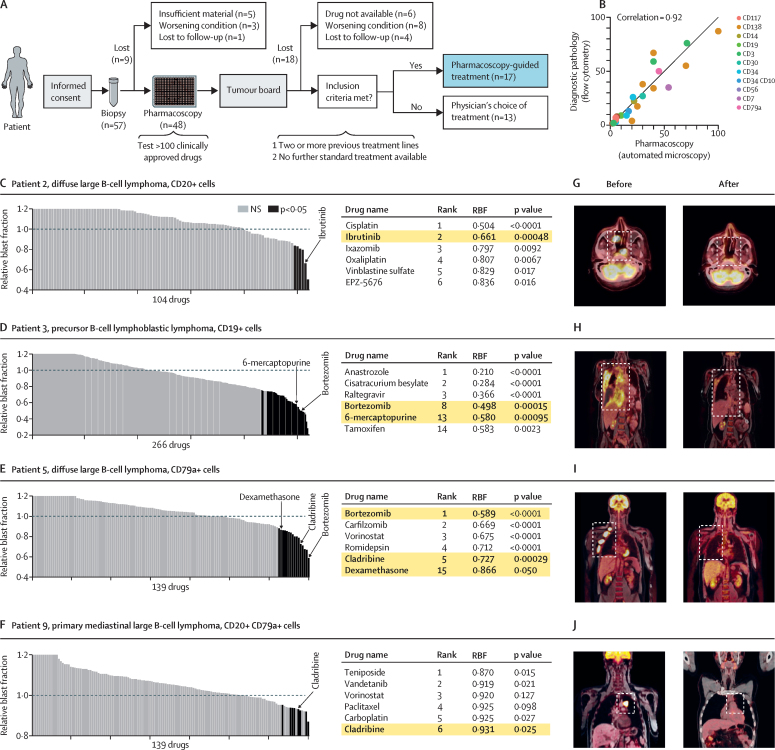
Study outline and representative responses to pharmacoscopy-guided treatments (A) Outline of the workflow of this study, including patient numbers (n) and reasons for patient dropping out of the study. (B) Comparison of percentage of marker-positive cells identified by flow cytometry-based diagnostic pathology in the clinic and by automated microscopy and single-cell image analysis in patients for which both datasets were available. (C–F) RBFs for all screened drugs for selected case studies of patients, with corresponding patient numbers, diagnoses, and used blast marker indicated. Bar graphs show drugs ranked by average RBF, indicating significant (dark grey) and non-significant (light grey) RBFs; values of RBF>1·2 are capped at 1·2. Horizontal line at value 1 indicates DMSO-control levels. Table showing top-hit drug names, ranks, RBF, and p values (two-tailed *t* test against controls); yellow highlights indicate selected drugs provided during pharmacoscopy-guided treatment. (G–J) PET-CT or PET-MRT scans corresponding to the patients described in (A–D), at the time of last relapse (before) and after pharmacoscopy-guided treatments (after). White outlined boxes indicate tumour foci. NS=not significant. RBF=relative blast fractions.

In the prospective study of the 57 patients with aggressive haematological malignancies, nine patients were not assessed by the board for reasons given in figure 2A. Of 48 patients who were assessed by the board, 18 were not included and 13 still had further treatment options, leaving 17 patients who met the inclusion criteria to receive pharmacoscopy-guided treatment (figure 2A). For these 17 patients, pharmacoscopy was always done on the same day as the biopsy procedure, and median time to report pharmacoscopy results back to clinicians was 5 days (IQR 2–8). The trial started on Sept 1, 2015, the censoring date for the interim analysis for all patients was Nov 11, 2016, and the median follow-up time was 7·6 months (IQR 4·5–8·7). The characteristics of the 17 patients who had pharmacoscopy-guided treatment are listed in the table. The 13 patients reviewed by the board that received treatments not guided by pharmacoscopy served as an observational cohort (appendix p 8). A comparison between the percentage of marker-positive cells measured from the same biopsies by clinical diagnostics-based flow cytometry, the current gold standard, and by pharmacoscopy revealed strong consistency between the two methods (*r*=0·92, p<0·0001; figure 2B).

**Table:** Characteristics, treatments, and clinical responses of the 17 patients receiving pharmacoscopy-guided treatment

	**Diagnosis**	**Age (years)**	**Previous treatment lines**	**Sample type**	**Clinical diagnostic mutations**	**Cell markers used**	**Pharmacoscopy-guided treatment**	**Overall response**	**Progression-free survival (weeks)**	**Ongoing response**
1	B-cell acute lymphoblastic leukaemia	23	5	Peripheral blood	NRAS, CDKN2A	CD10, CD34	Bortezomib	Partial response	5·3	No
2	Diffuse large B-cell lymphoma	69	7	Dissociated lymph node	MYD88, CDKN2A	CD20	Ibrutinib	Complete remission	42·0	No
3	Precursor B-cell lymphoblastic lymphoma	51	3	Pleural effusion	Not determined	CD19, CD20	Obinutuzumab, 6-mercaptopurine, bortezomib	Partial response	12·9	No
4	Peripheral T-cell lymphoma	56	4	Bone marrow	TP53	CD3	Ixazomib, lenalidomide, dexamethasone	Complete remission	22·6	No
5	Diffuse large B-cell lymphoma	29	2	Dissociated lymph node	No alterations detected	CD79a	Bortezomib, cladribine, dexamethasone	Complete remission	34·0	Yes
6	B-cell acute lymphoblastic leukaemia	29	2	Peripheral blood	FLT3, KRAS	CD20, CD34	Bortezomib, azacitidine	Complete remission	37·1	Yes
7	Diffuse large B-cell lymphoma	60	5	Dissociated lymph node	MYD88	CD19, CD20	Imatinib, ibrutinib, lenalidomide, obinutuzumab; fludarabine, cyclophosphamide*	Stable disease	37·3	Yes
8	Acute myeloid leukaemia	72	2	Peripheral blood	NRAS	CD34, CD117	Azacitidine	Complete remission	22·4	No
9	Primary mediastinal large B-cell lymphoma	27	6	Dissociated lymph node	No alterations detected	CD20, CD30, CD79a	Brentuximab vedotin, cladribine	Complete remission	34·7	Yes
10	T-cell lymphoblastic lymphoma	31	4	Peripheral blood	PIK3CA, FBXW7, NOTCH1	CD3	Bortezomib, cyclophosphamide, dexamethasone	Partial response	4·1	No
11	Acute myeloid leukaemia	72	3	Peripheral blood	NPM1, KRAS	CD34, CD117	Decitabine	Partial response	8·4	No
12	Diffuse large B-cell lymphoma	67	3	Lymph node	MYC	CD20, CD79a	Ibrutinib	Complete remission	21·9	Yes
13	Follicular lymphoma grade 3A	63	3	Skin biopsy	TP53	CD19, CD20, CD79a	Bortezomib, cladribine, dexamathasone	Complete remission	19·3	Yes
14	T-cell prolymphocytic leukaemia	40	2	Peripheral blood	No alterations detected	CD3	Venetoclax	Partial response	13·9	No
15	Acute myeloid leukaemia	76	4	Bone marrow	No alterations detected	CD34, CD117	Azacitidine	Partial response	3·6	Yes
16	Diffuse large B-cell lymphoma	53	3	Dissociated lymph node	TP53	CD19, CD79a	Pixantrone, idelalisib, obinotuzumab	Partial response	7·4	Yes
17	Diffuse large B-cell lymphoma	50	3	Bone marrow	TP53	CD19, CD20, CD79a	Azacitidine, panobinostat, atorvastatin	Stable disease	3·3	No

Data are provided for each patient (number 1–17). Patients 5, 6, and 9 could proceed to allogeneic stem-cell transplantation, patient 7 received CART-19 transfusion.

* Administered sequentially.

Pharmacoscopy-guided treatment regimens resulted in encouraging partial and complete remissions (table), documented as indicated by the type of malignancy, for example by PET-CT or PET-MRI for lymphoma. To visualise the workflow, we present data for the first four patients who had partial or complete response, as documented by PET-CT or PET-MRT. Patient 2, a 69-year-old man with diffuse large B-cell lymphoma, relapsed after seven lines of previous treatment. Lymphoma cells of the sample were resistant to most of the 104 drugs tested, and only six compounds had significant on-target effects ex vivo (figure 2C). Cisplatin and oxaliplatin were not regarded as feasible given the patient's history, age, and comorbidities; however, the Bruton's tyrosine kinase inhibitor ibrutinib had the second strongest ex-vivo efficacy (RBF 0·61, p=0·00048; figure 2C). PET-CT imaging on day 49 of ibrutinib treatment confirmed a complete remission for the patient (figure 2G). Good clinical responses have been reported for a small subset of patients with diffuse large B-cell lymphoma carrying *MyD88* mutations.^37^ Subsequent sequencing confirmed that patient 2 had a *MyD88* mutation (table). Patient 3, a 51-year-old women with precursor B-cell lymphoblastic lymphoma, had three lines of previous treatment, and was progressive after immunotherapy with the bispecific CD3–CD19 antibody blinatumomab (table). Cells isolated from a pleural effusion were tested by pharmacoscopy against a panel of 266 compounds in duplicate, which revealed significant ex-vivo sensitivity to the proteasome inhibitor bortezomib (RBF 0·498, p=0·00015) and the thiopurine 6-mercaptopurine (RBF 0·580, p=0·00095; figure 2D). 6-mercaptopurine and bortezomib were combined with anti-CD20 obinutuzumab. After 28 days PET-CT confirmed a partial response (figure 2H). For patient 5, cells from an excised lymph node were tested for 139 drugs (figure 2E). The patient achieved a complete remission (figure 2I) to a combination of the single strongest ex-vivo acting drug bortezomib (RBF 0·589, p<0·0001), with cladribine ranked fifth (RBF 0·727; p=0·00029) and dexamethasone ranked 15th (RBF 0·866; p=0·050; figure 2E). And after ex-vivo sensitivity to cladribine was measured for patient 9 (figure 2F), complete remission was observed with cladribine treatment in combination with CD30-targeted immunotherapy brentuximab vedotin (figure 2J). Data for patient 7, one of the two patients who did not respond to pharmacoscopy-guided treatment, is shown and further discussed in the appendix (p 4).

**Figure 3:**
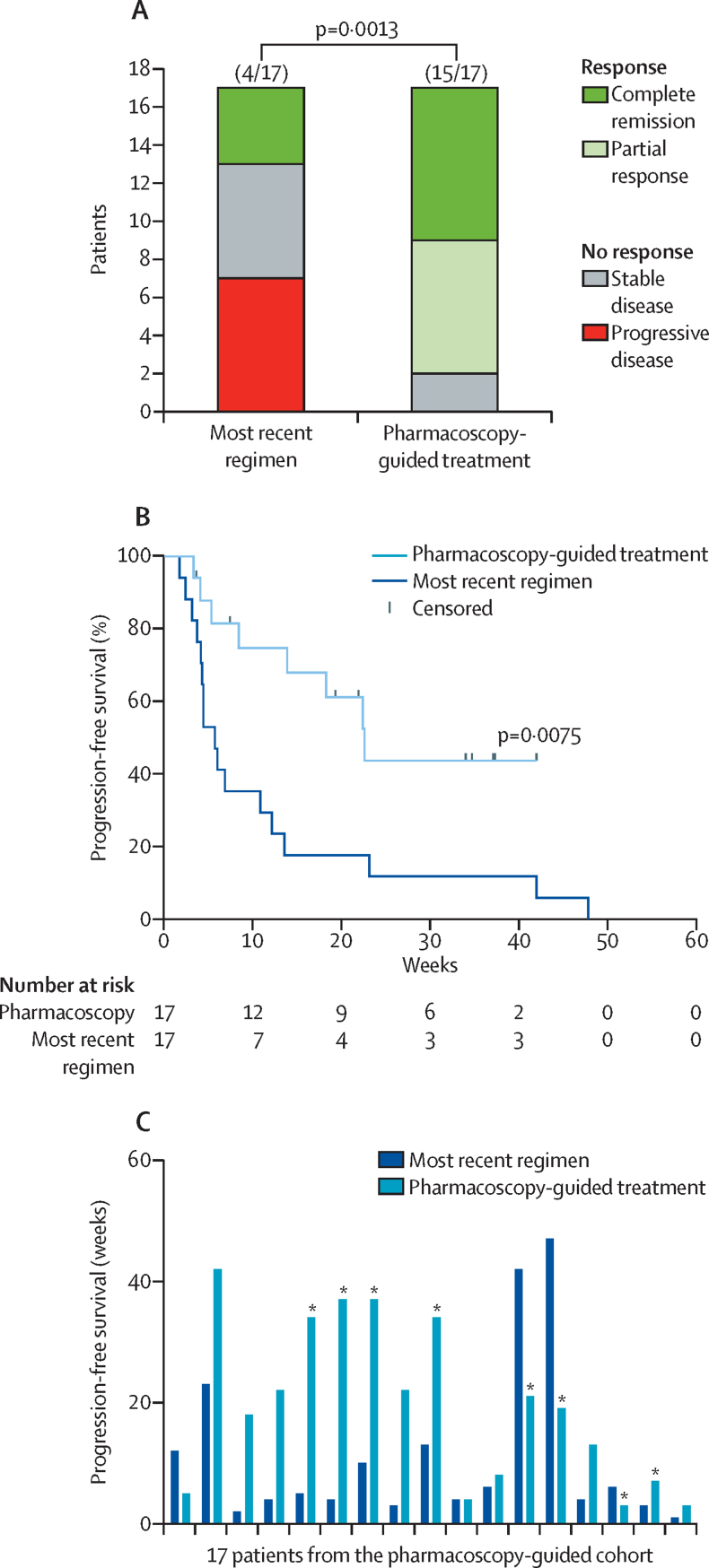
Overall response and progression-free survival with pharmacoscopy-guided treatment (A) Comparison of overall response with the most recent regimen and of pharmacoscopy-guided treatments for 17 patients with aggressive haematological malignancies. p value was calculated by McNemar's test for paired binomial data. (B) Kaplan-Meier plot showing progression-free survival with the most recent regimen and pharmacoscopy-guided treatments for 17 patients. (C) Progression-free survival with most recent regimen or pharmacoscopy-guided treatment per patient. *Ongoing response at time of analysis.

Overall response and progression-free survival of pharmacoscopy were compared with overall response and progression-free survival for the most recent regimen on which the patient had progressed. Four (24%) of 17 patients achieved an overall response with the most recent regimen compared with 15 (88%) of 17 patients who achieved an overall response with pharmacoscopy-guided treatment (odds ratio 24·38 [95% CI 3·99–125·4], p=0·0013; figure 3A). Five (38%) of 13 patients receiving standard salvage treatment based on physician's choice achieved an overall response (appendix p 8). Notably, none of the 17 patients receiving pharmacoscopy-guided treatments had progressive disease as best overall response, whereas seven patients had progressive disease in response to their most recent regimen (figure 3A). Furthermore, pharmacoscopy-guided treatments also led to a significantly improved median progression-free survival (22·6 weeks [95% CI 7·4–34·0]) compared with a median of 5·7 weeks (4·1–12·1) in the same patients with the most recent regimen (hazard ratio 3·14 [95% CI 1·37–7·22], p=0·0075; figure 3B). 12 (71%) of 17 patients had a progression-free survival ratio of 1·3 or higher (figure 3C). The null hypothesis was therefore rejected. Notably, eight (47%) of 17 patients that received pharmacoscopy-guided treatment regimens still had ongoing responses at the time of analysis (figure 3C), including patients 5, 6, and 9, who could proceed to allogenic stem-cell transplantation, and patient 7, who proceeded to CART-19 transfusion. Five (29%) of 17 patients received treatment regimens that included immunotherapy as part of their pharmacoscopy-guided treatment, potentially confounding our interpretation of the results. We therefore re-tested clinical response after exclusion of these five patients, which showed that both overall response (p=0·0002) and progression-free survival (p=0·025) remained significantly improved for pharmacoscopy-guided treatments compared with the most recent regimen. This reanalysis allowed us to exclude the possibility that the addition of antibody-based immunotherapies affected our interpretation of the results. Taken together, pharmacoscopy-guided treatment regimens demonstrated strongly improved clinical responses and survival benefit in an initial cohort of 17 late-stage patients with aggressive relapsed and refractory haematological malignancies.

**Figure 4:**
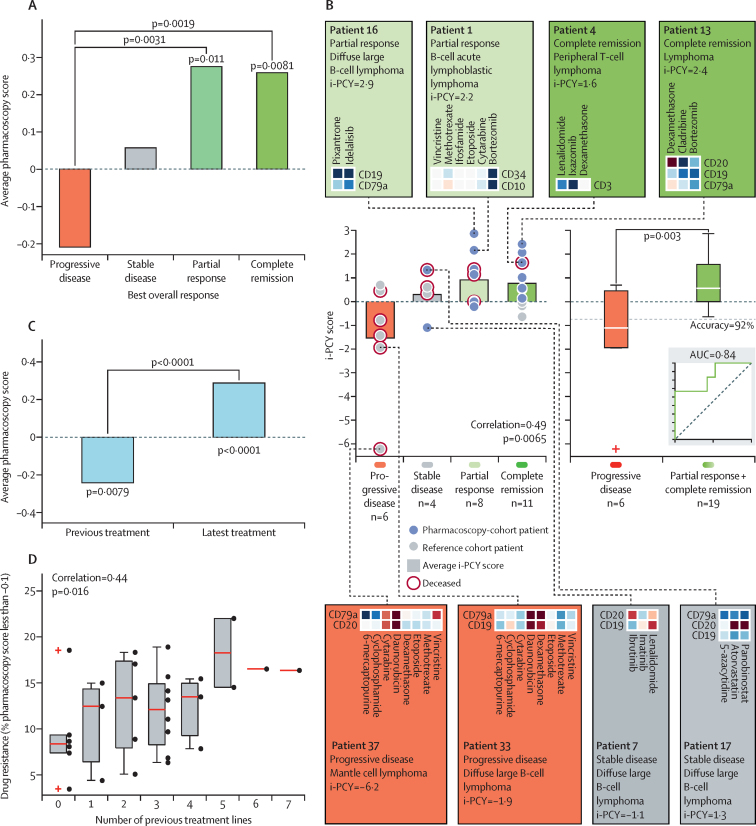
Pharmacoscopy and therapeutic response (A) Average pharmacoscopy scores in all patients per best overall response reveal negative scores associated with progressive disease. (B) i-PCY score per patient by best overall response in 29 patients. Individual dots correspond to individual patients. Bars show average i-PCY scores by overall response. Box and whisker plots show i-PCY scores for progressive disease and partial response and complete remission responses. Inset shows the corresponding ROC curve. Coloured boxes show pharmacoscopy data for all markers and drugs for selected patient; heat map colours range from dark red (iPCY<–1) to white (iPCY=0) to dark blue (PCY>1), see also the legend in the appendix (p 5). i-PCY=integrated pharmacoscopy. (C) Average pharmacoscopy scores. p values directly above bars indicate significant deviation from 0; p values of pairwise comparisons are indicated by corresponding connecting lines (A, C). (D) Box and whisker plots of the tested drugs to which ex-vivo resistance is observed by pharmacoscopy per number of previous treatment lines in 29 patients. Individual patient values are plotted as black dots next to the boxplots. ROC=receiver operating characteristics. Crosses indicate datapoints that do not fall between the whiskers.

To test whether chemoresistance measured by pharmacoscopy is predictive of poor clinical response we did a cluster analysis including the 17 patients receiving pharmacoscopy-guided treatment with the 12 observation cohort patients whose subsequent treatments were also tested ex-vivo before treatment initiation. Hierarchical clustering of the pharmacoscopy response profiles per patient and blast-markers as determined by clinical diagnostics, overlaid with the best overall response corresponding to drug and patient pairs, revealed extensive patient-to-patient variability in both the number and identity of drugs to which either chemoresistance or chemosensitivity was measured (appendix p 5). Similar indications displayed remarkable heterogeneity in response profiles, indicating an absence of characteristic ex-vivo responses for the tested indications in this partially heavily pretreated cohort. Hierarchical clustering repeatedly grouped drug classes with the same mode of action, including immunomodulatory drugs (thalidomide, lenalidomide, and pomalidomide), anthracycline chemotherapies (daunorubicin, doxorubicin, and valrubicin), and histone deacetylase inhibitors (belinostat, panobinostat, and vorinostat). The clustering further highlighted the diversity of treatments given to the patients. 30 unique drugs, distributed across the clustering, were tested by pharmacoscopy and subsequently administered to patients, enabling robust pan-treatment statistical analysis (figure 4, appendix p 5).

Further analyses demonstrated the association between ex-vivo chemoresistance and poor clinical outcome. First, plotting the average pharmacoscopy scores over all markers and drugs in relation to associated overall response to those drugs showed that treatments leading to progressive disease were associated with negative pharmacoscopy scores, whereas treatments leading to partial response or complete remission resulted in significantly positive pharmacoscopy scores (figure 4A). Second, the treatments to which the patient had relapsed before pharmacoscopy testing had on average negative pharmacoscopy scores (p=0·0079; figure 4C). Third, the percentage of tested drugs to which ex-vivo resistance was measured (at pharmacoscopy score less than −0·1) increased with the number of previous treatment rounds of each of the 29 patients (*r*=0·44; p=0·016; figure 4D). Similar significantly positive correlations were found when defining chemoresistance as pharmacoscopy scores of less than −0·2, less than −0·3, or less than −0·4. Patients who had received no or only one previous treatment line showed ex-vivo chemoresistance (a pharmacoscopy score less than −0·1) to 9% of tested drugs, whereas patients that had received five or more previous treatment lines showed ex-vivo chemoresistance to 17% of tested drugs (p=0·016; figure 4D).

Patient outcomes correlated positively with the integrated pharmacoscopy scores (*r*=0·49, p=0·0065; figure 4B). 18 (94%) of 19 responding patients (partial response and complete remission) had i-PCY scores between 0 to 3, whereas four (67%) of six patients with progressive disease, all of whom did not receive pharmacoscopy-guided treatments, had i-PCY scores in the negative range between −0·75 and −7. Patient treatments associated with high i-PCY scores combined drugs acting on-target on all tested blast markers, or combined neutral, ex-vivo acting drugs with ex-vivo on-target acting drugs. Conversely, patients responding with progressive disease as best overall response had treatments including drugs to which strong, ex-vivo chemoresistance was measured. One of two non-responding (stable disease) patients receiving pharmacoscopy-guided treatments had an i-PCY score of below −1, indicating that the pharmacoscopy test did not strongly support the final personalised treatment regimen for this non-responding patient, due to ex-vivo discordance depending on the used blast markers (figure 4B). Overall, the i-PCY score separated progressive disease from patients who had achieved a partial response and complete remission with a classification accuracy of 92% and an AUC of 0·84 (figure 4B).

## Discussion

This single-centre study shows technical feasibility of integrating automated microscopy-based, ex-vivo drug-response profiling for patients with aggressive haematological malignancies into clinical practice. The test-guided treatment regimens led to significantly longer progression-free survival and improved overall response in patients with various haematological malignancies compared with their most recent regimens, warranting further disease-specific clinical studies that include larger patient cohorts and randomised control groups.^38^ Although the trial did not include a randomised control group and had a relatively small cohort size of 17 patients, our results suggest that a wide array of working chemotherapeutics and targeted inhibitors already exist, which, in principle, are capable of breaking drug resistance even in multirefractory cancers, if the right drugs are selected at the right time for each individual patient. We found that an integrative combination of chemosensitivity of the leukaemic blasts and chemoresistance of the marker-negative, non-malignant cells predicted clinical response to first-line acute myeloid leukaemia treatment with the highest accuracy. Furthermore, the same readout guided selection of treatments associated with favourable clinical responses, and predicted both good as well as poor clinical responses. The positive relation observed between the number of previous treatment lines and ex-vivo drug resistance is intuitive, and might reflect acquired drug resistance as well as refractory disease being more resistant from disease onset.

Our investigation was designed as a prospective, non-randomised study in which every patient acted as their own control. This approach allowed us to assess the overall effect across heterogeneous diseases and treatment regimens; however, the absence of randomisation could have led to bias.^6^, ^38^ Future randomised trials testing pharmacoscopy-guided therapies versus physician's choice are therefore warranted, and should focus on individual disease entities.

Not all patients in our study had correlation between pharmacoscopy results and outcome, in particular one outlier patient (patient 10 in the retrospective acute myeloid leukaemia study). Identifying the causes for such outliers will thus require repetition with larger cohort sizes and integration with systematic molecular data. In our comparison of conventional genetics with response in the retrospective acute myeloid leukaemia cohort, our results matched those in previous studies.^39^

A benefit of pharmacoscopy resides in the analytical power derived from monitoring with computer-aided precision millions of individual single-cell drug responses, which combined with the ability to discriminate cell types allows us to score specific rather than general and averaged cytotoxic effects. Pharmacoscopy will likely be instructive for the personalised identification of clinically effective therapies for other malignancies beyond those tested here. The selection of personalised therapy by pharmacoscopy benefits from the ability to measure hundreds to thousands of drug exposures using small patient samples, in which each ex-vivo treatment includes healthy cell controls from the same patient sample. Pharmacoscopy detects cancer cells with fluorescently labelled antibodies against clinically used diagnostic markers, which means the test synergises with, and uses similar antibodies as, clinical flow cytometry-based diagnostics. Both microscopy and flow cytometry or optometry share the limitations of detection of cancer cells by antibody-based immunofluorescence, whereas pharmacoscopy allows for reduced sample processing and increased throughput and automation. Our results show that single-cell detection of blast markers by pharmacoscopy enables a clinically useful comparison of on-target and off-target cytotoxicity, while the minimal ex-vivo culturing of cells, and compatibility with clinical diagnostic markers, ensure fast and relevant feedback. Specifically, the platform allowed us to test 768 conditions for almost all of the 17 patient samples, returning results to the clinic within 5 days of receiving a sample. A crucial trade-off nonetheless remains between the number of different drugs, technical replicates, concentration ranges, timepoints, and drug combinations that can be tested from one biopsy. In that regard, the observation made in this study that ex-vivo testing of single treatments can aid selection of clinically beneficial combination treatments suggests that not every drug combination needs to be tested in combination ex vivo, thus allowing for larger drug panels to be tested; future studies are needed to further refine optimal, ex-vivo drug-testing regimens.

Comprehensive drug response profiles of individual people, as generated here, represent the outcome of interplay between various molecular parameters of the responding cells, including not only the genetic, proteomic, and metabolic state of the cells, but also the direct and indirect molecular interactions with other cells.^30^ We therefore hypothesise that such comprehensive drug-response profiles can offer novel functional insight into the underlying health status of an individual, with potentially wide-ranging implications in preventive and participatory medicine. Given the fast throughput of the method, both experimentally and analytically, future studies can include higher patient numbers that will be of great interest to investigate the translatability of pharmacoscopy further.

Pharmacoscopy provided useful treatment-guidance in an initial late-stage patient cohort, warranting further investigation in larger and indication-specific clinical trials. It is likely that the approach will synergise well with molecular profiling techniques such as genomics and proteomics for personalised treatment identification. Such studies could lead to improved patient treatment and be a useful route to mechanistic elucidation of clinically relevant genotype-to-phenotype relationships.
