# Supplementary material for: Image-based ex-vivo drug screening for patients with aggressive haematological malignancies: interim results from a single-arm, open-label, pilot study
Source: Lancet Haematol. 2017 Nov 15;4(12):e595–606. doi: 10.1016/S2352-3026(17)30208-9 (PMC5719985; doi:10.1016/S2352-3026(17)30208-9)
Supplement: Supplementary appendix [file mmc1.pdf]

THE LANCET
Haematology

Supplementary appendix

This appendix formed part of the original submission and has been peer reviewed. We post it as supplied by the authors.

Supplement to: Snijder B, Vladimer G I, Krall N, et al. Image-based ex-vivo drug screening for patients with aggressive haematological malignancies: interim results from a single-arm, open-label, pilot study. *Lancet Haematol* 2017; published online Nov 15. http://dx.doi.org/10.1016/S2352-3026(17)30208-9.

http://dx.doi.org/10.1016/S2352-3026

Supplementary Appendix to:

Image-based *ex vivo* drug screening for patients with aggressive haematological malignancies: interim results from a single arm, open-label, pilot study

Berend Snijder*, Gregory I Vladimer*, Nikolaus Krall, Katsuhiro Miura, Ann-Sofie Schmolke, Christoph Kornauth, Oscar Lopez de la Fuente, Hye-Soo Choi, Emiel van der Kouwe, Sinan Gültekin, Lukas Kazianka, Johannes W Bigenzahn, Gregor Hoermann, Nicole Prutsch, Olaf Merkel, Anna Ringler, Monika Sabler, Georg Jeryczynski, Marius E Mayerhoefer, Ingrid Simonitsch-Klupp, Katharina Ocko, Franz Felberbauer, Leonhard Müllauer, Gerald W. Prager, Belgin Korkmaz, Lukas Kenner, Wolfgang R. Sperr, Robert Kralovics, Heinz Gisslinger, Peter Valent, Stefan Kubicek, Ulrich Jäger, Philipp B. Staber‡, Giulio Superti-Furga‡

* Contributed equally
‡ Contributed equally

Supplementary Figure 1	Fresh vs frozen pharmacoscopy comparison	Page 2
Supplementary Figure 2	PCY results per AML retrospective patient	Page 3
Supplementary Figure 3	Prospective patient #7: example of a non-responder	Page 4
Supplementary Figure 4	Full prospective pharmacoscopy results clustered	Page 5
Supplementary Table 1	Details for AML retrospective patients	Page 6
Supplementary Table 2	ANOVA analysis of AML retrospective data	Page 7
Supplementary Table 3	Details of 13 patients receiving physician's choice	Page 8
Legend to Supplementary Table 4	Details for the tested drugs	Page 9

http://dx.doi.org/10.1016/S2352-3026

Figure S1

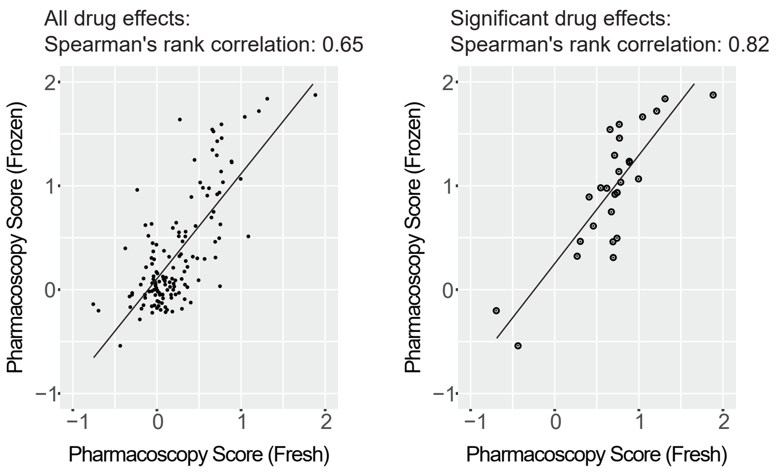

Figure S1. Comparison of pharmacoscopy results from the same biopsy, fresh and frozen. Scatter plots with pharmacoscopy results per drug, comparing the results from part of the biopsy that was frozen (y-axis) with part of the biopsy that was processed immediately (x-axis). Left scatter plot shows all drugs, right scatter plot shows only those drug results that deviated significantly ($P<0.05$) from the control distribution.

Figure S2

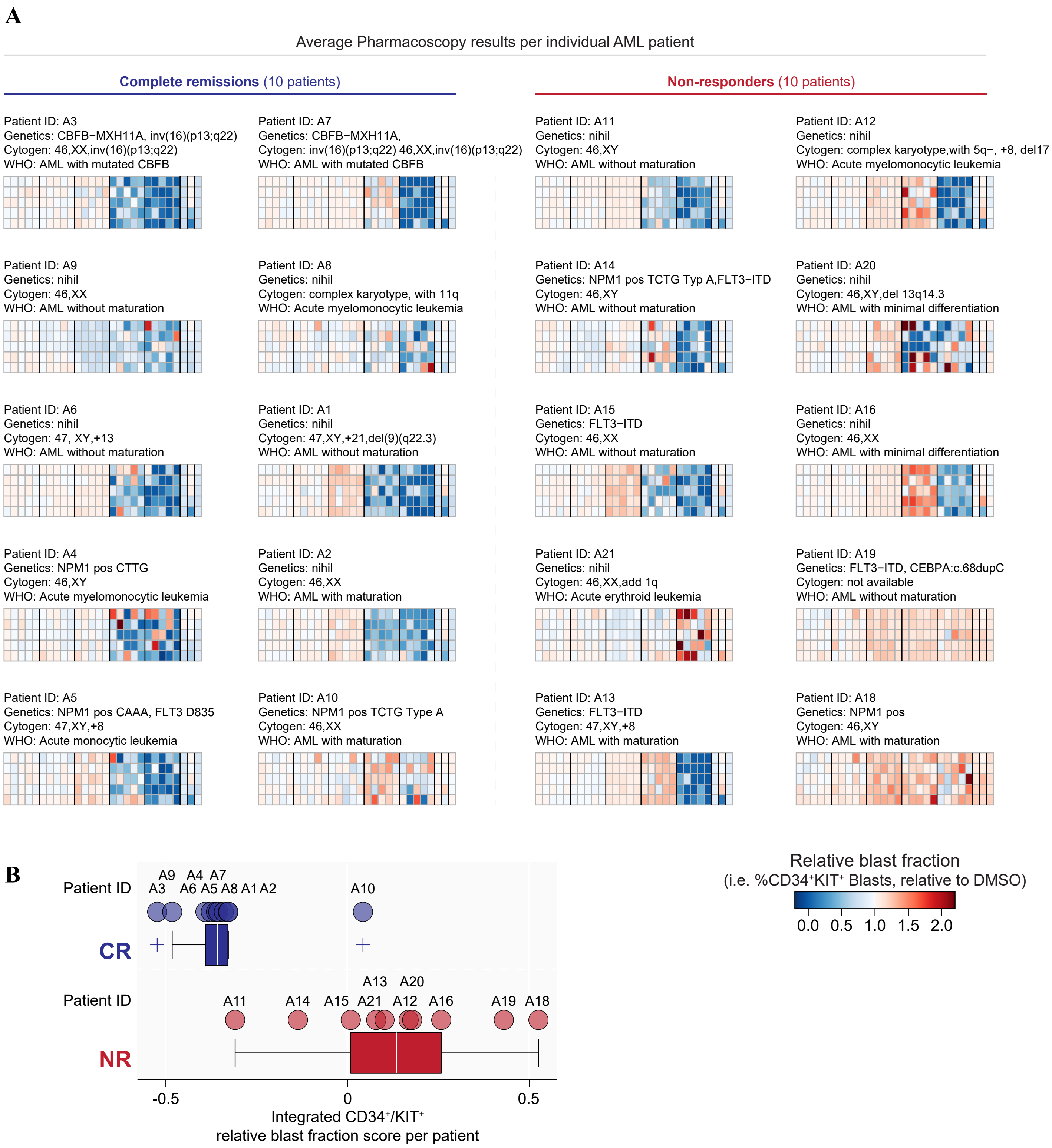

Figure S2. Per-patient results from the retrospective AML study. (A) Heatmaps depict DMSO-relative percentages of $CD34^{+}/KIT^{+}$ cells per patient and per drug combination, as in Figure 1C. Clinical characteristics per patient are given above each heatmap (as in Table S1). (B) Box-and-whisker plot of the integrated $CD34^{+}/KIT^{+}$ scores as in Figure 1H, indicating individual patient identifiers for each data point. (A) Average over four technical replicates per patient are shown.

http://dx.doi.org/10.1016/S2352-3026

Figure S3

Patient #7, Diffuse large B-cell lymphoma, Dissociated lymph node, Stable disease.

A

CD19+ cells

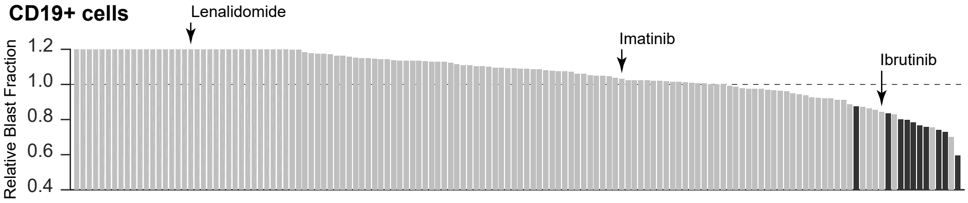

Rank	Significance	RBF	Drug
#1	p=1.3e-07	0.596	Daunorubicin
#2	p=0.38	0.701	Clofarabine
#3	p=0.0022	0.731	Trametinib
#4	p=0.0098	0.740	Etoposide
#13	p=0.99	0.844	Ibrutinib
#54	p=0.59	1.033	Imatinib
#122	p=1	1.286	Lenalidomide

B

CD20+ cells

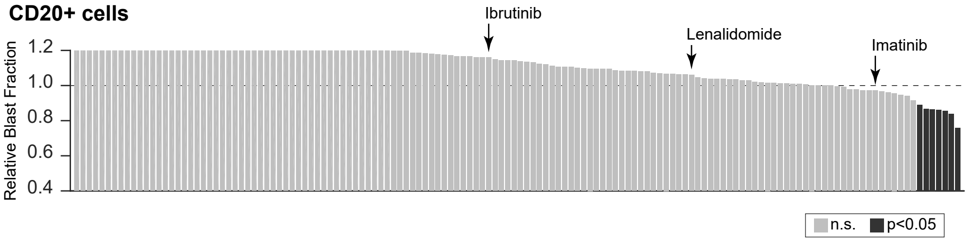

Rank	Significance	RBF	Drug
#1	p=0.0011	0.760	Pemetrexed
#2	p=0.0062	0.837	Volasertib
#3	p=0.012	0.855	Gefitinib
#4	p=0.0074	0.860	JQ1
#14	p=0.34	0.972	Imatinib
#43	p=0.70	1.061	Lenalidomide
#75	p=0.94	1.159	Ibrutinib

Figure S3. Patient #7, who did not respond to Pharmacoscopy-guided treatment. Relative blast fractions (RBF) over all screened drugs based on the markers CD19 and CD20 from patient #7. (**A**) Bar graphs show drugs ranked by average CD19-RBF, indicating significant (dark) and non-significant (light) RBFs; Values of RBF>1.2 are capped at 1.2. Horizontal line at value 1 indicates DMSO-control levels. Table inserts on the right show data for selected and top drug hits. Yellow highlighted rows indicate drugs that were part of the pharmacoscopy-guided treatment for this patient. (**B**) Same as (**A**), but for RBF values calculated based on $CD20^{+}$ cells. Stable disease was determined through physical assessment of the tumor mass over the course of the treatment scheme.

http://dx.doi.org/10.1016/S2352-3026

Figure S4

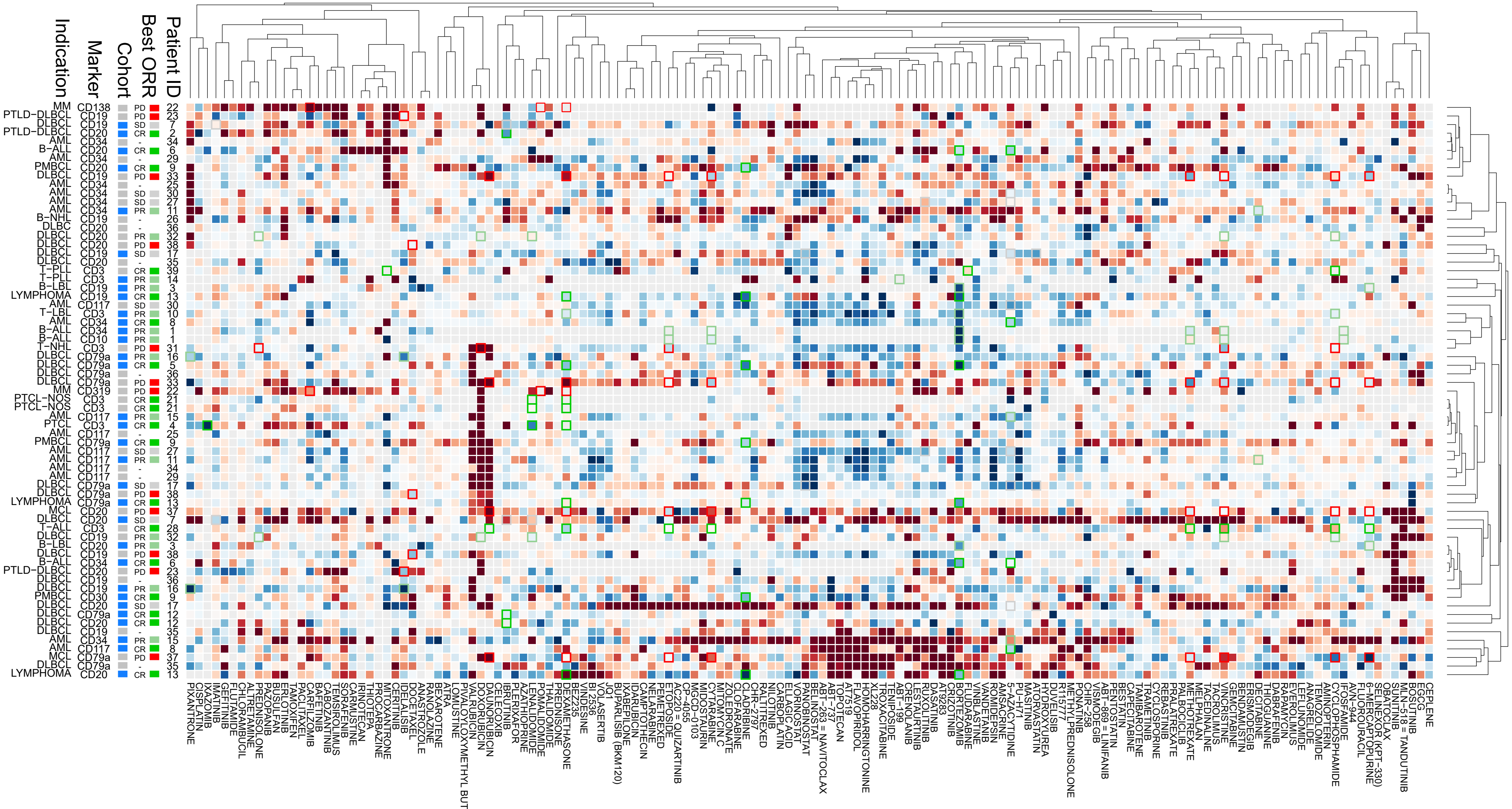

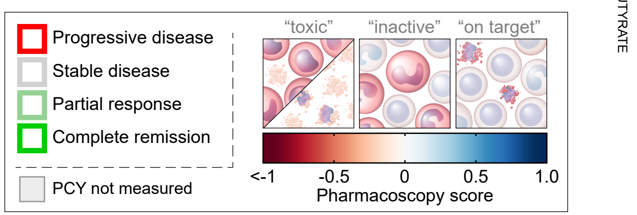

Figure S4. Clustering of pharmacoscopy results per patient, marker, and drug. (A) Clustering of PCY results per drug (columns) and patient sample / marker combination (rows) over all patients. Note that the number and identity of molecular markers used to identify cancer cells for each patient were adapted to clinical diagnostics results and can thus vary per patient. Colors indicate PCY scores, and selected boxes are outlined to indicate the corresponding ORR to that patient/drug combination (see legend).

Supplementary Table 1

Patient ID	Cohort	Age at diagnosis	Date of diagnosis	% blasts in bone marrow at diagnosis	Date of start of therapy	Date remission check (bone marrow)	% blasts in bone marrow after 1st induction	Blast: CD34+	Blast: CD33+	Blast: CD117+	Molecular Mutation	Cytogenetic	Molecular/ Cytogenetic Risk*	WHO classifcation
A_1	CR	35	5/25/2009	>90%	5/27/2009	6/26/2009	1%	+	-	+	no marker detected	47,XY,+21,del(9)(q22.3)	Intermediate	AML without maturation
A_2	CR	29	4/22/2009	~80%	4/25/2009	6/9/2009	1%	+	-	+	no marker detected	46,XX	Intermediate	AML with maturation
A_3	CR	42	5/11/2009	90%	5/14/2009	6/19/2009	1%	+	-	+	CBFB-MXH11A, inv(16)(p13;q22)	46,XX,inv(16)(p13;q22)	Favorable	AML with mutated CBFB
A_4	CR	42	1/28/2010	70%	1/30/2010	3/2/2010	2%	-	+	+	NPM1 pos CTTG	46,XY	Favorable	Acute myelomonocytic leukemia
A_5	CR	45	11/2/2009	90%	11/3/2009	12/4/2009	2%	-	+	-	NPM1 pos CAAA, FLT3 D835	47,XY,+8	Intermediate	Acute monocytic leukemia
A_6	CR	53	12/30/2009	75-80%	12/31/2009	2/9/2010	1%	+	+	+	no marker detected	47, XY,+13	Intermediate	AML without maturation
A_7	CR	64	4/9/2010	90%	4/10/2010	5/18/2010	n/a	+	+	+	CBFB-MXH11A, inv(16)(p13;q22)	46,XX,inv(16)(p13;q22)	Favorable	AML with mutated CBFB
A_8	CR	56	11/24/2010	~70%	11/27/2010	12/28/2010	below detection limit	+	-	-	no marker detected	complex karyotype, with 11q	Unfavorable	Acute myelomonocytic leukemia (secondary)
A_9	CR	64	5/27/2009	~80%	5/29/2009	7/3/2009	1%	+	-	+	no marker detected	46,XX	Intermediate	AML without maturation
A_10	CR	60	3/23/2010	n/a	3/30/2010	5/4/2010	below detection limit	50%+	+	+	NPM1 pos TCTG Type A	46,XX	Favorable	AML with maturation
A_11	NR	64	3/3/2009	90%	3/5/2009	3/30/2009	~85%	+	+	+	no marker detected	46,XY	Intermediate	AML without maturation
A_12	NR	54	10/12/2010	~80%	10/23/2010	11/8/2010	~40%	30-40% +	>90% +	30-40% +	no marker detected	complex karyotype, with 5q-, +8, del17	Unfavorable	Acute myelomonocytic leukemia
A_13	NR	55	6/24/2013	>90%	6/25/2013	7/25/2013	25%	+	+	+	FLT3-ITD	47,XY,+8	Unfavorable	AML with maturation
A_14	NR	54	3/1/2010	>90%	3/4/2010	4/13/2010	~90%	-	-	+	NPM1 pos TCTG Typ A,FLT3-ITD	46,XY	Unfavorable	AML without maturation
A_15	NR	51	3/21/2013	>80%		4/24/2010	~85%	-	+	+	FLT3-ITD	46,XX	Unfavorable	AML without maturation
A_16	NR	71	2/20/2009	~80%	2/25/2009	4/3/2009	~90%	+	-	+	no marker detected	46,XX	Intermediate	AML with minimal differentiation
A_18	NR	76	1/2/2013		1/12/2013	2/12/2013	diffuse infultrate	-	-	+	NPM1 pos	46,XY	Favorable	AML with maturation
A_19	NR	65	1/15/2014	90%		2/25/2014	~20%	-	+	-	FLT3-ITD, CEBPA:c.68dupC	not available	Unfavorable	AML without maturation
A_20	NR	59	2/26/2013	50%	3/23/2013	5/7/2013	~30%	+	+	+	no marker detected	46,XY,del 13q14.3	Intermediate	AML with minimal differentiation
A_21	NR	44	9/6/2013	30%	09_2013	10_2013	not completed	n/a	n/a	n/a	no marker detected	46,XX,add 1q	Intermediate	Acute erythroid leukemia

Table S1: Clinical details of each patient from which biobanked bone marrow samples were assessed in the AML retrospective assay. Patient information is organized by their corresponding anonymized patient identifier. Age at and date of diagnosis, percent blasts per sample before and after treatment, duration of therapy, diagnostic marker status, molecular biology, and WHO classification are given. "Nihil" indicates no molecular markers were identified. Patients classified clinically as non-responders are in red, those who achieved stable and complete remission are in blue. * Molecular/Cytogenetic Risk classifications are according to Leukemia (2014) 28, 50–58; doi:10.1038/leu.2013.236.

Supplementary Table 2

Source	Sum Sq.	d.f.	Mean Sq.	F	Prob>F (P-value)
**Cohort**	14.70	1	14.70	100.86	**0**
Cytarabine	0.49	4	0.12	0.84	0.497
**Daunorubicin**	78.04	2	39.02	267.73	**0**
Etoposide	1.13	4	0.28	1.94	0.1021
Cohort × Cytarabine	0.13	4	0.03	0.22	0.9247
**Cohort × Daunorubicin**	2.68	2	1.34	9.19	**0.0001**
Cohort × Etoposide	0.85	4	0.21	1.46	0.2135
Cytarabine × Daunorubicin	0.96	8	0.12	0.82	0.5851
Cytarabine × Etoposide	1.24	16	0.08	0.53	0.9312
Daunorubicin × Etoposide	1.51	8	0.19	1.30	0.2409
Cohort × Cytarabine × Daunorubicin	0.60	8	0.08	0.51	0.8464
Cohort × Cytarabine × Etoposide	1.35	16	0.08	0.58	0.9007
Cohort × Daunorubicin × Etoposide	0.79	8	0.10	0.68	0.7102
Cytarabine × Daunorubicin × Etoposide	3.34	32	0.10	0.72	0.8785
Error	201.41	1382	0.15		
Total	309.22	1499			

Table S2: Details of the analysis of variance for the AML retrospective study. Table providing further details on the test statistics of a 3-way ANOVA analysis with (1) cohort, (2) cytarabine concentration, (3) daunorubicin concentration, and (4) etoposide concentration as factors and patients as random effects. Significant observations are highlighted in bold.

Supplementary Table 3

Diagnosis	Age at time of biopsy	Prior treatment lines	Sample Type	Genetics	Physician's choice	Best ORR	PFS (weeks)	Ongoing response
PTCL-NOS	77	1	Dissociated Lymph Node	TCRB pos., TCRG neg.MUM1 pos.	Lenalidomide, Dexamethasone	CR	46.1	Y
MM	71	0	Dissociated Lymph Node	IGK neg., IGH neg.	Carfilzomib, Dexamethasone, Elotuzumab, Pomalidomide	PD	1.7	N
PTLD-DLBCL	56	3	Dissociated Lymph Node	BRAF, PTEN, TP53	Rituximab, Idelalisib	PD	4.1	N
ALCL ALK neg	47	2	Dissociated Lymph Node	n.d.	Lenalidomide, Dexamethasone	PR	8.9	N
AML	62	0	Peripheral Blood	JAK2 pos.	Ruxolitinib And 5-Azacytidine	SD	17.0	N
T-ALL	40	0	Peripheral Blood	BCR/ABL neg. 46XY.	Methotrexate, Dexamethasone, Cyclophosphamide, Vincristine, Daunorubicin, Cytarabine,6-Mercaptopurine, Etoposide	CR	30.1	Y
AML	78	0	Bone marrow	n.d.	5-Azacytidine	SD	6.4	N
T-NHL	64	0	Peripheral Blood	n.d.	Prednisolone, Vincristine, Cyclophosphamide, Doxorubicin, Etoposide	PD	9.9	N
DLBCL	74	0	Dissociated Lymph Node	MYC translocation neg., MYD88 neg.	Lenalidomide, Prednisolone, Vincristine, Cyclophosphamide, Doxorubicin	PR	24.3	Y
DLBCL	76	1	Dissociated Lymph Node	n.d.	Methotrexate, Dexamethasone, Cyclophosphamide, Vincristine, Daunorubicin, Cytarabine, 6-Mercaptopurine, Etoposide	PD	13.3	N
Mantel cell lymphoma	52	2	Dissociated Lymph Node	n.d.	Methotrexate, Dexamethasone, Cyclophosphamide, Vincristine, Daunorubicin, Cytarabine, 6-Mercaptopurine, Etoposide	PD	2.6	N
DLBCL	32	3	Dissociated Lymph Node	P53 pos., MYD88 neg., MYC translocation (-), BCI translocation (-)	Docetaxel, Penbrolizumab	PD	3.7	N
T-PLL	76	1	Bone marrow	TCL-1 translocation neg.	Fludarabine, Cyclophosphamide, Mitoxantrone	CR	11.1	N

Table S3. **Details of the 13 patients receiving physician's choice treatment** Table providing characteristics, treatments, and clinical responses, of 13 patients that received treatment of physician's choice after pharmacoscopy-testing. For abbreviations see legend of Table 1.

http://dx.doi.org/10.1016/S2352-3026

Table S4. **Details of the 139 drugs that form the most-used drug panel in this study** Table providing details on the 139 drugs that make up the drug panel most often used in this study. CHEM_NAME: chemical name; MW: molecular weight; SIMPLIFIED DRUG CLASS contain a high level simplified drug annotation; DRUGBANK ANNOTATION: contains annotations computationally obtained from https://www.drugbank.ca/; CHEBI ANNOTATION: contains annotations computationally obtained from https://www.ebi.ac.uk/chebi/.

Please find the full Table S4 online at *The Lancet Haematology*.
